# Computational Design of Catalysts with Experimental Validation: Recent Successes, Effective Strategies, and Pitfalls

**DOI:** 10.1021/acs.jpcc.4c04949

**Published:** 2024-10-17

**Authors:** Hajar Hosseini, Connor J. Herring, Chukwudi F. Nwaokorie, Gloria A. Sulley, Matthew M. Montemore

**Affiliations:** Department of Chemical and Biomolecular Engineering, Tulane University, New Orleans, Louisiana 70118, United States

## Abstract

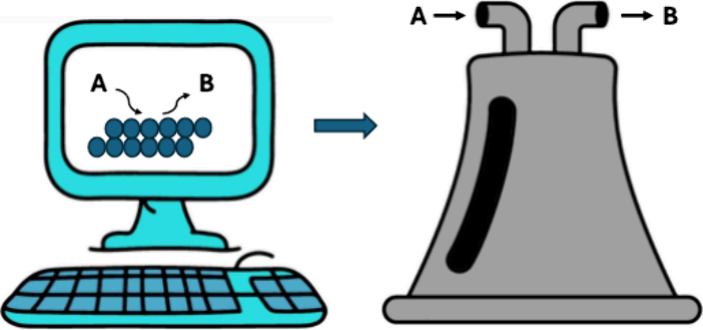

Computation has long proven useful in understanding heterogeneous catalysts and rationalizing experimental findings. However, computational design with experimental validation requires somewhat different approaches and has proven more difficult. In recent years, there have been increasing successes in such computational design with experimental validation. In this Perspective, we discuss some of these recent successes and the methodologies used. We also discuss various design strategies more broadly, as well as approximations to consider and pitfalls to try to avoid when designing for experiment. Overall, computation can be a powerful and efficient tool in guiding catalyst design but must be combined with a strong fundamental understanding of catalysis science to be most effective in terms of both choosing the design methodology and choosing which materials to pursue experimentally.

## 1 Introduction

Heterogeneous catalysis plays a pivotal role in achieving energy-efficient and selective molecular transformations.^1,2^ Both current-day industries and potential clean energy technologies rely critically on efficient catalysis; therefore, there is a critical need to innovate and develop new, cost-effective, and efficient catalysts to propel the future of these technologies.^3^ Traditionally, new catalysts have been developed through trial-and-error or intuition. However, there have been significant recent advances in computation-driven design and screening for catalysis.

Understanding and applying structure–property relationships in nanoscale solids for heterogeneous catalysis is challenging due to their complexity. A particular challenge is developing structural models for presumed active sites for calculations. Given the inherent complexity in the surface structures of nanoscale materials, as well as possible morphological, compositional, and structural changes caused by the reactive atmosphere, identifying or predicting active sites under steady-state or dynamic operation is often difficult. Furthermore, these catalysts are often structurally heterogeneous and complex, featuring various facets, defects, metal–support interfaces, etc.^4^ Complex approaches can be applied to help address these challenges;^5^ however, these complex methods are typically computationally intensive and thus difficult to apply for screening and design. Thus, screening and design often focus on simpler principles to estimate catalytic properties like activity, selectivity, and stability, in order to screen a materials space of various compositions and structures and discover improved catalysts.^6,7^

Most computational catalysis studies focus on understanding existing systems, developing fundamental insight into trends, or developing new design strategies.^8^ When using computation to design new systems with the intention to perform experimental validation, different considerations are required. For example, a certain level of material stability is crucial when experimental validation is desired but is not necessarily needed when attempting to understand fundamental trends in chemical properties. Thus, it is crucial to carefully consider the goals of a specific study and decide which considerations are necessary to meet those goals.

Here, we briefly review some recent studies that computationally design catalysts and chemically reactive surfaces and experimentally validate those predictions. When validating computational predictions, it can be challenging to ensure that the experimental comparison between materials is fair and comprehensive, and that the experiments are probing a similar material and surface structure as that proposed by the computation. Since most experimental validations only consider a few materials and detailed atomic-scale characterization is difficult—particularly under reaction conditions—it is possible for the experiment to probe a material that is different in some important aspect from the computational structure, and yet agree with the computational prediction purely by serendipity. Thus, while we do not discuss the experimental validation in great detail in most cases, the specifics of the experiments can be crucial in determining whether the validation is truly effective. We focus on recent studies that design metal alloy catalysts, but we also discuss a few studies of other types of heterogeneous catalysts. In addition to these success stories, we also discuss various strategies for computational catalyst design and some common pitfalls. Our goals are to demonstrate that computation is clearly useful for driving catalyst design in many cases, and to give a broad overview of effective strategies when designing new catalysts with the intent to validate experimentally.

## 2 Recent Studies with Computational Design and Experimental Validation of Catalysts

### 2.1 Descriptor-Based Approach

Several recent studies have used a small number of adsorption energies and/or transition state energies to design catalysts that were experimentally realized. These quantities act as descriptors, or simple proxies that allow estimation of the catalytic performance.

**Figure 1:**
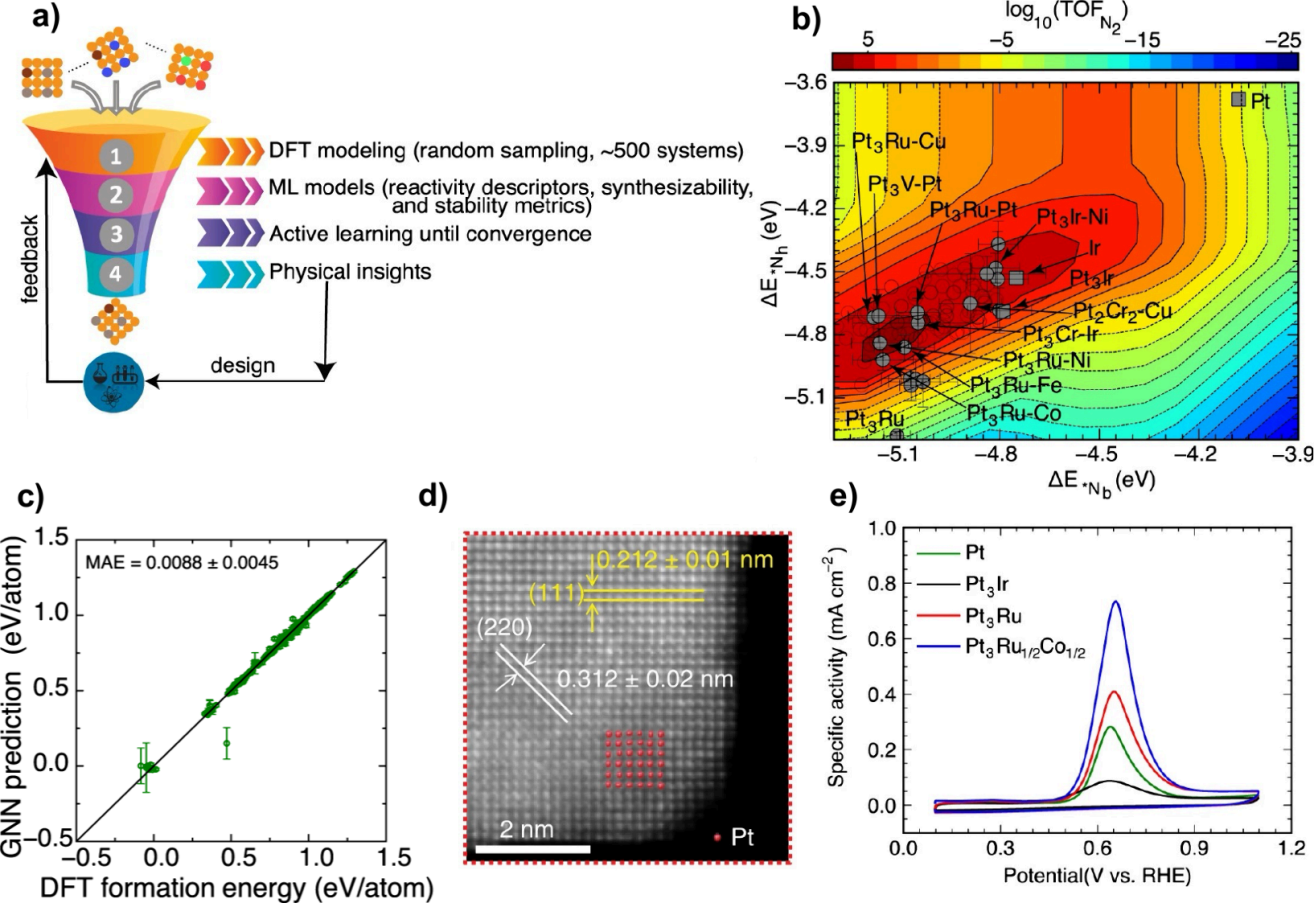
(a) Implementing an active learning process to expedite the discovery of catalytic materials. (b) Mapping activity at 0.3 V vs reversible hydrogen electrode (RHE), showcasing promising ternary Pt alloy electrocatalysts. Pt, Ir, and Pt_3_Ir electrocatalysts are depicted by solid square markers. Open markers represent systems with theoretical activity surpassing Pt_3_Ir but failing stability and synthesizability filters. (c) 10-fold final model of graph neural network for predicting formation energy. (d) Enlarged HAADF-STEM image showing the atomic structure of the surface region of the Pt_3_Ru_1/2_Co_1/2_ alloy. (e) Specific activity comparisons for the Pt, Pt_3_Ir, Pt_3_Ru, and Pt_3_Ru_1/2_Co_1/2_ nanocubes. Adapted with permission from ref (9). Copyright 2023 The Authors under a CC-BY 4.0 license, published by Springer Nature.

Many of these studies are based on the volcano-plot paradigm, wherein the binding strength of one (or a few) simple adsorbates is used to estimate the rate, with the idea that the binding strength should be neither too strong nor too weak. For example, a volcano plot for NH_3_ electrooxidation was developed and applied based on the bridge- and hollow-site N adsorption energies (see Figure 1).^9^ This plot correctly predicted that Pt_3_Ir and Ir are more active than Pt. The plot was then used to screen for Ir-free trimetallic electrocatalysts featuring {100}-type site motifs by forecasting site reactivity, surface stability, and catalyst synthesizability descriptors. Next, Pt-alloy cubic nanoparticle catalysts supported on reduced graphene oxide were synthesized, characterized, and tested for electrochemical performance. High-angle annular dark-field-scanning transmission electron microscopy (HAADF-STEM, Figure 1d) and X-ray diffraction (XRD) were used to confirm the structures. Cyclic voltammetry was then performed on all of the samples using the same electrolyte. These experiments showed that, as predicted, Pt_3_Ru_1/2_Co_1/2_ demonstrated superior mass activity toward ammonia oxidation as compared to Pt, Pt_3_Ru, and Pt_3_Ir catalysts (Figure 1e). The design approach was further validated by comparing activity trends including additional trimetallic alloys (Pt_3_Ru_1/2_Fe_1/2_ and Pt_3_Ru_1/2_Ni_1/2_) and showing that the computationally predicted trends matched the experimentally determined trends. Volcano plots have also been used to screen a series of metal-on-metal bimetallics for nitrite reduction based on the N_2_, NH_3_, and N adsorption energies.^10^ Based on this screening, Pd-on-Au nanoparticles were synthesized and showed a relatively high selectivity toward N_2_ as compared to pure Pd.

Volcano plots have also been used in recent studies to discover novel heterogeneous catalysts for alkane dehydrogenation. For example, the C and CH_3_ adsorption energies were chosen as computationally facile descriptors for ethane dehydrogenation.^11^ Pt_3_Sn displayed better ethylene selectivity and coke resistance than Pt, but limited activity. To search beyond the volcano plot, a decision map was created with a score of 1 for catalysts with higher selectivity than Pt and a higher turnover frequency than PtSn_3_, and a score of 0 otherwise. Final candidates were chosen based on the assumption that the crystal structures in the inorganic crystal structure database are naturally stable, and based on price. The approach was verified by DFT calculations for all reaction intermediates and transition states on Ni_3_Mo, Ni_3_Cr, and NiAl_3_. Ni_3_Mo/MgO was synthesized and outperformed Pt/MgO, consistent with the theoretical prediction. The Ni_3_Mo/MgO catalyst achieved an ethane conversion of 1.2%, three times higher than the 0.4% conversion for Pt/MgO under the same reaction conditions. The ethylene selectivity of Ni_3_Mo/MgO started at 66.4% and increased to 81.2% after 12 h, while Pt/MgO began at 75.2% and slightly rose to 79.3%. Methane was the main byproduct observed on both catalysts. Likewise, DFT calculations combined with machine learning were used to design selective, stable, and synthesizable heterogeneous catalysts for propane dehydrogenation.^12^ DFT calculations were performed for the reaction pathway from propane to propyne on an initial set of surfaces. The strongest pair of descriptors were then identified with statistical analysis, and CH_3_CHCH_2_ and CH_3_CH_2_CH were selected based on existing chemical understanding of propane dehydrogenation. The resulting volcano map aligned with existing experimental data. Similar to above, a decision map was created based on similarity to Pt. Screening of a series of bimetallic alloys identified promising candidates, including some without noble metals, and NiMo was chosen for experimental testing. Accordingly, a NiMo/Al_2_O_3_ catalyst was synthesized and showed better performance over Pt/Al_2_O_3_ in terms of its selectivity, activity, and stability over time.

Other studies have used a few simple descriptors with strategies other than the volcano plot to estimate performance. For example, DFT calculations of the adsorption energy difference between O_2_ and PO_4_^3–^ were used to screen Pt alloy surfaces for oxygen reduction reaction activity and phosphoric acid resistance.^13^ Several Pt_*x*_Cu catalysts were synthesized based on these calculations, and Pt_2_Cu was found to display good mass activity and phosphoric acid resistance. In another study, Cu-based single-atom alloys (SAAs) for propane dehydrogenation were screened by calculating the transition state energy for initial C–H scission, which is usually the rate-determining step. Rh_1_Cu was chosen as it has a low barrier for propane activation, comparable to a pure Pt surface. Since the Cu host is expected to facilitate propylene desorption and be resistant to coking, screening by choosing a low activation energy was expected to result in a catalyst that can activate propane but does not adsorb undesired carbonaceous intermediates too strongly. Surface science and reactor experiments validated the computational results, showing that RhCu/SiO_2_ SAA catalysts are more active and stable than Pt/Al_2_O_3_.^14^

In addition to the work on metal alloys, the descriptor-based approach has also been applied to other types of catalysts. For example, PCN-250(Fe_2_M) metal–organic frameworks (MOFs) were modeled with DFT as potential catalysts for light alkane C–H bond activation with N_2_O as the oxidant.^15^ The calculations focused on the barrier for N_2_O activation on MOFs that are synthesizable such as PCN-250(Fe_3_, Fe_2_Mn, Fe_2_Co, and Fe_2_Ni). The predicted calculation trend of PCN-250(Fe_2_Mn) ∼ PCN-250(Fe_3_) > PCN-250(Fe_2_Co) > PCN-250(Fe_2_Ni) based on the N_2_O activation barrier height was confirmed using experiments. DFT calculations have also been used to craft effective single-atom-doped Ga_2_O_3_ catalysts for propane dehydrogenation to illustrate how the Lewis acid–base interaction can disrupt the typical volcano-shaped activity curve, enabling superior catalytic performance compared to the peak of the volcano.^16^ In the absence of the Lewis acid–base interaction, the formation energy of H adsorption on top of O was used as the descriptor. In the presence of the Lewis acid–base interaction, the formation energy of H–H coadsorption at the M-O site was used. Pt_1_–Ga_2_O_3_ and Ir_1_–Ga_2_O_3_ were predicted to display good performance, which was verified by experimental synthesis and testing of Pt–Ga_2_O_3_ and γ-Al_2_O_3_-supported Ir–Ga_2_O_3_ catalysts.

In another study, ion-doped CoP was screened for alkaline hydrogen evolution, primarily based on the H adsorption free energy,^17^ with symbolic regression used to create a model for the H adsorption free energy. Based on these predictions, Al-CoP, V-CoP, and Mo-CoP were synthesized. Structure and morphology characterization were performed via scanning electron microscopy (SEM), TEM, elemental mapping, XRD, and X-ray photoelectron spectroscopy (XPS). These techniques showed the structure and morphology of the catalyst and its surface, confirmed successful ion doping into the surface, and showed that pure CoP had the expected structure while doping did not generate any new crystalline phases. Electrochemical measurements showed that, consistent with the predictions, Al-CoP, V-CoP, and Mo-CoP gave lower overpotentials (75 mV, 83 mV, and 134 mV, respectively) than undoped CoP (206 mV). The exchange current density and turnover frequencies also showed improvements upon doping.

### 2.2 Design Using Calculations of Multiple Reaction Steps

Some recent studies have shown that DFT calculations of reaction energetics of multiple elementary steps are useful for designing catalysts. For example, a range of bimetallic and trimetallic alloys were explored to investigate potential enhancements for current Ag catalysts in ethylene epoxidation.^18^ First, Ag/Cd, Ag/Zn and Ag/Cu were identified as potential bimetallic candidates based on examination of the reaction energetics, with Ag/Cu being chosen for further study. Then, DFT calculations of the full reaction mechanism and microkinetic modeling were used to investigate six metal combinations, namely, Ag/Cu with Hg, Cd, In, Tl, Pb, and Zn. Ag/CuTl, Ag/CuPb, and Ag/CuCd were predicted to show similar activity and better selectivity compared to Ag/Cu. Experimental synthesis and testing showed the highest activity for Cu- and Pb-modified catalysts and the highest selectivity for Ag/CuCd, in agreement with the predictions.

DFT has also been used to examine dual-atom catalysts anchored on nitrogen-doped graphene with varying coordination setups for electrocatalytic nitrate reduction to ammonia.^19^ Multiple adsorption energies and the reaction energies for multiple steps were used to screen 80 dual-atom catalysts. Based on this screening, the full reaction pathway was calculated on a Cu_2_ dual-atom catalyst, which was then experimentally synthesized and validated.

### 2.3 Screening Focused on Stability

**Figure 2:**
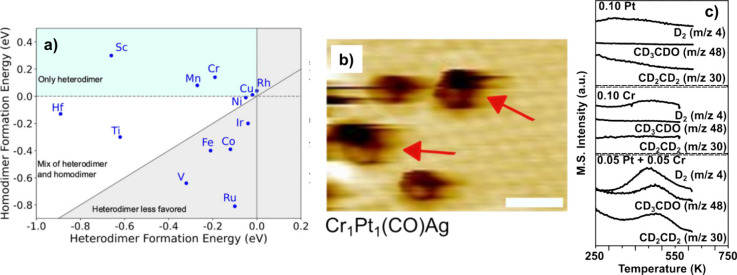
(a) Screening for a metal to add to the PtAg SAA to form a dual-atom alloy, based on the homodimer and heterodimer formation energies. Cr was chosen for further study. (b) Subsequent scanning-tunneling microscopy images showing the formation of PtCr pair sites in Ag(111). (c) TPD studies showing that PtCrAg can convert ethanol, while PtAg and CrAg cannot. Adapted with permission from ref (20). Copyright 2023 American Chemical Society.

Some research has focused on predicting stability for guiding experimental development of new catalysts and reactive surfaces. For example, several combinations were screened to discover stable dual-atom alloys based on calculated heterometallic and homometallic dimer formation energies (see Figure 2).^20,21^ Of the combinations that were predicted to be thermodynamically stable, Pt_1_Cr_1_Ag was chosen as a candidate with potentially favorable chemical properties for ethanol activation. Based on this, trimetallic PtCrAg was synthesized using a Ag(111) single crystal, as well as bimetallic CrAg and PtAg as controls. PtCrAg displayed reactivity toward ethanol while the bimetallics did not, as rationalized by DFT calculations of ethanol dehydrogenation on these surfaces. Applying surface science techniques on well-defined surfaces can be a strong validation of the atomic-scale structure of computationally designed active sites, as also recently shown for PdSnAg surfaces.^22^

In a computational-experimental screening process on bimetallic catalysts, Pd was used as a benchmark for H_2_O_2_ synthesis.^23^ In the screening process, the formation energy of each bimetallic phase was calculated to determine the most stable crystal structures. Alloys with densities of states similar to Pd were expected to exhibit catalytic properties comparable to Pd. Experimental synthesis and testing of the promising candidates for H_2_O_2_ direct synthesis revealed that four of them (Ni_61_Pt_39_, Au_51_Pd_49_, Pt_52_Pd_48_, and Pd_52_Ni_48_) indeed displayed catalytic properties comparable to Pd. Notably, the Pd-free Ni_61_Pt_39_ alloy exhibited a 9.5-fold enhancement in cost-normalized productivity compared to Pd. In a study on doped CoP, the stability was assessed with the cohesive energy as a screening filter.^17^ Molecular simulations provided additional evidence that the designed systems would retain their thermal stability and structural integrity. In a study on doped Ga_2_O_3_, ab initio molecular dynamic simulations were used to test the stability of slab surface geometries and further dual atom-doped surface models.^16^

### 2.4 Leveraging Machine Learning for Screening

Machine learning methods can be used as part of screening in several ways. For example, they can efficiently predict descriptor values, can aid in determination of effective descriptors, or can incorporate experimental data.

Machine learning is often used to increase the efficiency of screening by increasing the speed of predicting descriptor values and was used for this purpose in some of the studies discussed above. For example, the ammonia oxidation study noted above used graph neural networks trained on ab initio data to predict site reactivity, surface stability, and catalyst synthesizability descriptors.^9^ Additionally, connections between reactivity descriptors and the machine-learned features of a site motif were elucidated with post hoc interpretation techniques to understand the superior performance of Pt_3_Ru-M (M: Fe, CO, or Ni) electrocatalysts. As another example, statistical analysis was used as an efficient method to search beyond the volcano plot for alkane dehydrogenation.^11,12^

Some recent studies have used experimental data in conjunction with machine learning or a combination of DFT and machine learning to design novel catalysts. For example, symbolic regression developed using 18 known oxide perovskite catalysts was used for discovering an appropriate descriptor for oxygen evolution reaction activities.^24^ The value of μ/*t*, where μ and *t* are the octahedral and tolerance factors, was found to be the most balanced descriptor in terms of complexity and accuracy, and can be directly used in material design without the need for additional DFT calculations. After using this framework for screening, new oxide perovskites were synthesized experimentally and were found to be among the materials with the highest specific activities. Furthermore, experiments had the same trend as that derived from μ/*t*. In a separate study, a smaller collection of experimentally measured catalytic activities and selectivities along with data mining from a large DFT database was used to identify important reaction steps in ethanol reforming.^25^ First, a machine learning model was trained for prediction of transition-state energies. Linear regression models for experimental catalytic activity and selectivity were established, utilizing DFT transition-state and reaction energies as the features. Based on this, the reforming activity of Pt/Mo_2_N was predicted to be three times greater than pure Pt with equally good selectivity.^26^ Experimental findings from both temperature-programmed desorption (TPD) and high-resolution electron energy loss spectroscopy (HREELS) suggested that the reaction of ethanol on the Pt/Mo_2_N monolayer surface exhibited similar selectivity and reaction intermediates to that on Pt(111), aligning with the predictions.

## 3 Design Strategies

As is evident from the above examples, several strategies can be employed for the rational design of catalysts with high activity, selectivity, and/or stability. In general, design strategies could range from simple descriptor-based approaches to more complex and computationally expensive techniques such as kinetic Monte Carlo simulations or ab initio thermodynamics. In this section, we give an overview of several broad strategies that could be used in catalyst design, along with some recent, illustrative examples.

### 3.1 Activity and Selectivity

In the simplest cases, descriptors such as the adsorption energy have seen substantial success as predictors of catalytic activity.^27^ These descriptors are often closely related to scaling relationships (i.e., linear correlations) between adsorption energies, which can reduce the dimensionality of a complex design problem.^28^ In some cases, the rate-descriptor relationship is described reasonably well by a volcano plot, which is a manifestation of the Sabatier principle.^29^ Specifically, the adsorption energy of a simple intermediate has been used to predict catalytic activity in a wide variety of systems, including combustion reactions^30^ and propane dehydrogenation.^12^ This is a particularly valuable approach for high-throughput screening; for example, a model which predicted adsorption energies for a variety of species on transition metal catalysts was used to rapidly screen approximately 10^7^ unique surface sites on 10^6^ unique surfaces for a number of industrially relevant reactions including ammonia synthesis, hydrogen evolution and oxygen reduction.^31^

Other descriptor-based approaches such as an activation energy or electronic structure parameter have been used to screen catalysts for a variety of reactions. For example, the initial activation energy in propane dehydrogenation was used to screen Cu-based SAAs for propane dehydrogenation, as noted above.^14^ Simply minimizing this activation energy would likely lead to coking if screening a broad variety of materials, but this design principle was effective because Cu will tend to mitigate coking. Further, the d-band center of transition metals is well-known to correlate linearly with energies of adsorption in some cases and has been used as a descriptor in many systems.^32^ Recently, the Co d_*z*_^2^ center in Co porphyrin catalysts was found to correlate with the O_2_ binding energy and was used to predict the activity for H_2_O_2_ synthesis.^33^ A model which was used to predict multiple adsorption energies of species on transition metal surfaces was also used to identify catalysts which break scaling relationships in the context of methane steam reforming.^34^ Further, a simple descriptor consisting of the number of d electrons and the electronegativity was found to accurately predict catalytic performance for the nitrogen reduction reaction.^35^

As discussed in Section 2, machine learning is often used in conjunction with simple descriptors to avoid the computational cost of quantum mechanical calculations. These approaches often feature trade-offs between accuracy, generality, and interpretability.^36,37^ Generally, machine learning is expected to accelerate descriptor prediction for nearly any descriptor if screening a broad materials space,^9^ or if a suitable methodology is chosen.^38^

As compared to simple descriptors, microkinetic modeling (MKM) generally provides an improvement in accuracy and transferability across different systems. Mean-field MKM is the most common approach, in which kinetics are calculated by breaking a reaction into elementary steps, each with their own power-rate law.^39^ Compared to the descriptor-based approach, this typically requires significantly more calculations of energetics in order to calculate the activation energies and rate constants, but generally requires fewer assumptions about scaling relationships and the operant mechanism. In particular, descriptors are most commonly developed by assuming linear relationships between energetics, and these relationships introduce additional, non-negligible error on top of the DFT energetics. MKM is most often used to understand a specific catalyst, as has been done for formic acid decomposition on Pt catalysts.^40^ However, MKM has also been used for catalyst design; for example, for alloy catalysts for ethylene epoxidation as discussed in Section 2.2.^18^ Even more accurate than MKM (but more computationally demanding) is kinetic Monte Carlo, which allows for nonuniformity in the distribution of reactants on surfaces and their configurations in different active sites.^41^ This typically requires additional DFT calculations to predict how adsorbate stabilities and rate constants depend on the local environment, such as different sites or with different nearby adsorbates. Even more intensive is first-principles molecular dynamics, which is very difficult to use in catalyst design as it uses time steps on the order of femtoseconds while catalytically relevant elementary steps generally occur on the order of nanoseconds to milliseconds.

**Figure 3:**
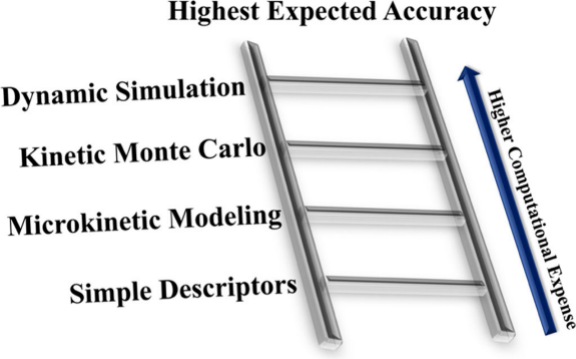
Jacob’s ladder of expected accuracy for catalytic activity prediction. In general, higher levels of accuracy feature more intensive computational requirements, and screening multiple materials becomes increasingly infeasible. This relationship assumes that each method is based on accurate energetics.

Overall, descriptor-based approaches are computationally convenient and relatively simple to understand. However, they may be limited by the underlying assumptions, particularly when only a few descriptors are used, and may not capture all relevant aspects of the reaction when applying them to new materials. For example, using a single adsorption energy as a descriptor may not be effective when scaling relations are broken.^29^ Mean-field MKM, kinetic Monte Carlo, and molecular dynamics are typically expected to be more accurate, but at an increasingly higher computational cost. More sophisticated methods can be very challenging to apply to a single catalyst, much less for screening multiple materials. Further, all of these techniques still require simplified models of the real system in nearly all cases in addition to relying on DFT which has its own inherent errors. Thus, the appropriate design strategy should be chosen with care, usually based on some previous understanding of the reaction and materials. Hierarchical approaches may be useful to quickly eliminate poor performers and focus effort on promising candidates. Figure 3 shows a Jacob’s ladder for catalytic activity, but the progression is not necessarily smooth or straightforward: methods that are expected to be less accurate could in fact be more accurate depending on the system, the quality of the underlying energetics, and the details of the model being used. Furthermore, these are purely computational predictions, and introducing experimental data in various ways can potentially improve the accuracy.

### 3.2 Stability

Much like the trade-offs between accuracy and computational cost seen in predicting catalytic activity, there are various techniques for forecasting stability, from simple and intuitive stability tests to advanced global optimization methodologies. There are many processes to consider when studying catalyst stability, including material rearrangement or degradation, coking/poisoning, sintering, etc. Here, we focus on strategies for predicting whether a given catalyst surface configuration is stable with respect to rearrangements such as surface segregation or phase segregation. While stability tests based on phonon spectra or molecular dynamics are useful for some materials,^42^ these methods focus more on dynamic stability, and we do not discuss them in detail here as they do not generally give insight into the thermodynamic stability of alloy surfaces, which generally rearrange on longer time scales. It is most important to determine stability under reaction conditions, but vacuum stability is often used as a simpler proxy, and the validity of this approach depends strongly on the system.

**Figure 4:**
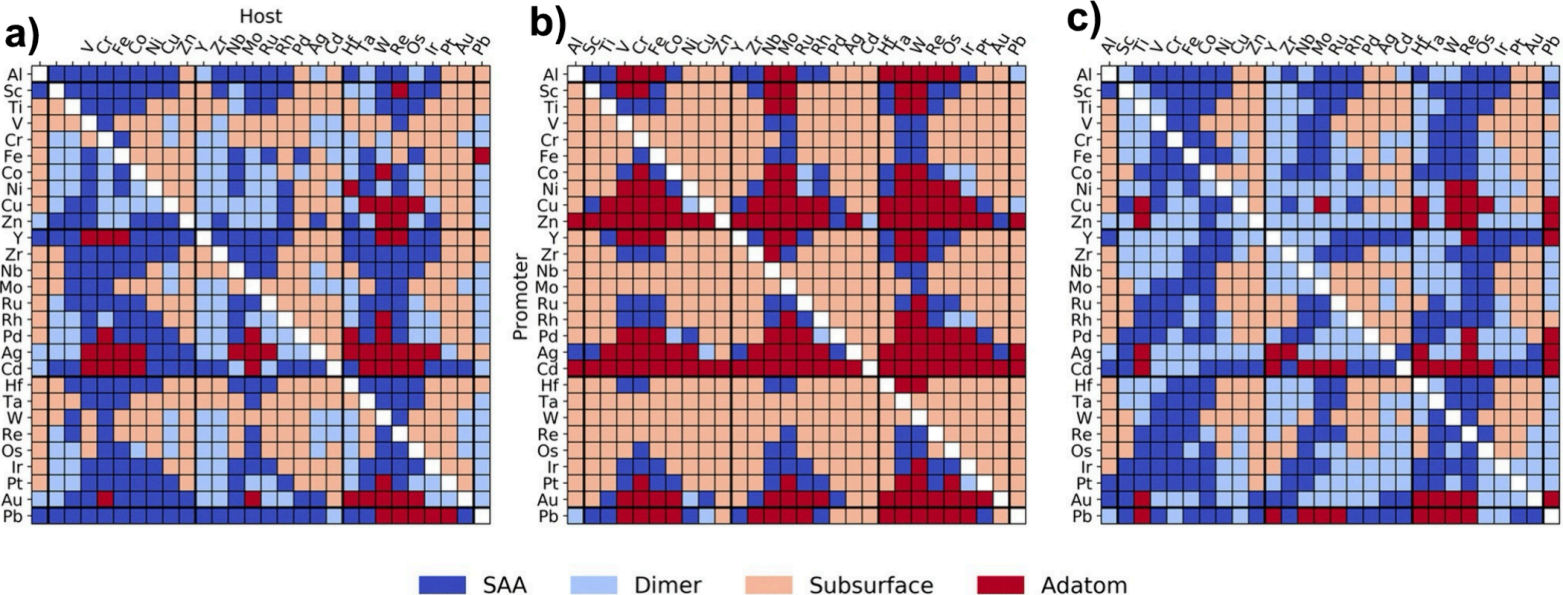
Most stable geometry (indicated by the color) for dilute binary surface alloys without adsorbed species, calculated by (a) DFT data, (b) a bond counting model, and (c) hybrid kernel ridge regression. Reprinted with permission from ref (44). Copyright 2020 Springer Nature.

Several studies have screened SAAs for their stability against clustering by calculating dimer formation and aggregation energies, both via DFT and machine learning models (see Figure 4).^43−45^ One such approach predicted favorable formation of Pt and Pd SAAs as well as a tendency for Co and Fe to aggregate in Cu SAAs, both of which have been experimentally confirmed.^45^ An analogous approach using dimer formation energies was used to predict dual-atom alloy stabilities and identify which dual-atom alloys are likely to be synthesizeable.^20^ Specifically, plotting the heterodimer versus homodimer formation energy helped to identify cases where the desired heterodimer is predicted to be more energetically favorable than either single-atom sites or homodimers. These relatively simple stability tests leverage what is already known about SAAs and their stability.

The surface segregation energy can also be used for stability prediction, as seen in the case of Rh-doped SAAs.^14^ This energy is a measure of how thermodynamically favorable it is for a dopant atom to occupy a surface site compared to a bulk or subsurface site. This can be calculated both in the vacuum and in the presence of adsorbates, which often significantly affect the result. As a broader example, the segregation energy was predicted using a fitted model for a range of surfaces of Pt-, Ir-, Pd-, and Rh-based SAAs.^46^ This model, which was trained on periodic slabs, was able to be transferred to SAA nanoparticles, predicting segregation energy with a similar level of accuracy. In a separate study, an “element centered fingerprint” representation of SAA surfaces was used to predict surface segregation energies for a variety of Ag-, Au-, and Cu-based SAAs both with and without CO.^47^ Overall, basic stability tests such as the segregation energy work well in simpler structures like SAAs and in cases where the likely mechanisms for rearrangement are well-known.

Beyond the relatively simple tests of stability discussed above, techniques such as ab initio thermodynamics have been developed to study material structure as a function of temperature and pressure.^48^ Based on DFT calculations, one can calculate the surface free energy for a range of configurations, where the configuration with the lowest surface free energy is the most thermodynamically stable at a given set of conditions and is thus predicted to be experimentally observed. This assumes that kinetic barriers do not prevent the system from achieving its most favorable state. As an example, this approach was used to predict the phase stability of PtO_2_ over a wide range of temperatures (0–600 K) and pressures (0–51 GPa). Calculations showed β-PtO_2_ was most stable at ambient pressure but would undergo a phase transition to α-PtO_2_ at higher (∼51 GPa) pressure.^49^ Similarly, ab initio thermodynamic analysis was used to evaluate IrO_2_ stability under oxygen evolution conditions while using the oxygen 2p-band center as a predictor of activity.^50^ This approach was also used to determine the stability of IrO_*x*_ structures which were identified via an active learning algorithm.^51^

A more challenging technique, global optimization, seeks the absolute lowest energy configuration across a system’s entire potential energy surface (PES). Predicting stability becomes more difficult when numerous intermediates or large, complex structures are involved. A brute force approach to this problem would involve creating many geometries and performing calculations to find the most energetically favorable. However, this method is subject to user bias if done manually and can become computationally infeasible if an exhaustive search is performed due to the vast number of candidate structures. While those strategies can be useful in many cases, in other cases it is beneficial to use global optimization techniques that can search for the global minimum of a PES without exhaustively evaluating individual candidates.^52^ Recently, these kinds of approaches have been used to predict a wide range of geometries including adsorbates on Rh surfaces,^52^ carbon products on CuZn catalysts,^53^ Cu(111) surfaces under electrochemical conditions,^54^ and Pt_n_ clusters.^55,56^

Overall, stability testing ranges from simple tests of likely or representative structures based on an understanding of the systems under study to fully automated global optimization. Because the more complex and accurate methods are computationally intensive, when screening many catalysts the most appropriate method must be chosen with care to give useful insight while remaining computationally feasible.

## 4 Approximations and Pitfalls

Computational design of catalysts holds great potential, as discussed in Section 2, but essentially always requires simplification of the system under study. Understanding the approximations made, as well as potential errors and pitfalls, can help improve design. We discuss many of the most important approximations and pitfalls here, roughly grouped by how easy they are to address. We note that many of the approximations can be addressed either in a simpler but less accurate way, or in a more complex but more accurate way, making this grouping somewhat arbitrary. We focus on physical approximations of catalytic systems, rather than the errors of DFT itself for predicting properties.

### 4.1 Approximations and Pitfalls That Are Easily Addressable

This section explores approximations and pitfalls that are often easy to address simply by making suitable choices when modeling the system.

One potential pitfall in computational design is the inaccurate modeling of surface reconstructions. Many metal surfaces are known to undergo reconstructions in their clean surface or in the presence of adsorbates, wherein the surface atoms assume a structure different from those in the bulk.^57^ Many bare metal surface reconstructions have been well characterized (see Table 1 for some of the most commonly encountered reconstructions). For example, the (100) surfaces of Au, Ir, Rh, and Pt undergo quasi-hexagonal reconstructions that are often modeled as (5 × 1) structures.^58,59^ The superstructure cell of Ir(100) is relatively small, whereas the unit cells of Pt(100) and Au(100) are much larger.^59^ More complex surfaces, such as stepped surfaces, can also reconstruct, as seen for Au(511);^60^ there is less work characterizing these surfaces and the precise reconstruction is not known for all surface facets of all metals. When modeling a surface with a known reconstruction, using the reconstructed structure is expected to improve accuracy unless there is reason to expect that the reconstruction is lifted due to the temperature, presence of adsorbed species, or material preparation.

**Table 1:** Some Commonly Encountered Reconstructions for Low-Index Metal Surfaces

Surfaces	Reconstruction
Au(110), Pt(110), Ir(110)	(1 × 2) missing-row reconstruction^61^
Au(111)	(22× herringbone pattern^62^
Au(100), Pt(100), Ir(100), Rh(100)	≈(5 × 1) quasi-hexagonal reconstruction^58,63,64,59^

Reconstruction—or lifting of reconstructions—of metal surfaces can occur in the presence of adsorbates, as metal atoms can rearrange to accommodate the adsorbate species.^65^ For instance, the “herringbone” (√3 × 22–rec) reconstruction of the Au(111) surface transforms into a striped (1 × 22) pattern in the presence of oxygen produced through the dissociation of nitrogen dioxide.^66^ Copper surfaces exhibit various oxygen-induced reconstructions, including a c(2 × 2) missing row reconstruction of Cu(100)^67^ and (n × 1) (n = 4, 3, 2) reconstructions for Cu(210).^65^ Carbon atoms can induce a double clock reconstruction of the fcc-Co(111) surface.^68^ Other examples include CO on Pt(100),^69^ S on Fe(110), S on Fe(110),^70^ and C on Ni(001).^70^ If the adsorbate-induced reconstruction for a particular case is known, it should be applied when relevant to more accurately represent the surface behavior. For example, studying a high coverage of adsorbed atomic O on many metal surfaces is less representative of the experimental system than studying the appropriate oxygen-induced reconstruction.

Another factor that is sometimes overlooked during computational design of catalysts is the stability of the surface configuration, ideally under the relevant experimental conditions. While it is convenient to computationally study a series of materials with the same configuration, assuming a surface structure for a given overall composition can lead to an incorrect model. Thus, selecting the appropriate stability tests that are relevant to the material under consideration is crucial. For example, SAAs are most likely to lose their desired structure (isolated single atoms) via clustering or diffusion of the dopant to the bulk/subsurface. Previous studies have suggested that adsorbed species can often stabilize the dopant on the surface and prevent movement to the subsurface.^71,47^ This suggests that the favorability of clustering, often measured by dopant dimerization energies under vacuum conditions, is likely the most useful initial stability test for SAAs, although as discussed above more complex strategies can be more accurate.

Another key consideration in computational catalyst design is the need to differentiate between potential and free energies. Specifically, the potential energy is calculated at 0 K (i.e., without entropy corrections), does not account for gas-phase pressure, and most often does not include zero-point energy corrections, which are typically important when breaking bonds involving H.^72^ Consequently, using solely potential energies to understand catalytic operation can be misleading, and free energies are generally more useful. Typically, the largest difference between potential and free energies is that gas-phase species become more stable (in terms of free energies) at higher temperature, due to the greater entropy of the gas phase. However, energetics for surface reactions can also be significantly affected by free energy corrections.

Relatedly, caution is needed when using a set of energetics to identify kinetically relevant steps or states, or to predict rates or catalytic trends. For example, assuming that the reaction step with the highest potential energy barrier is the rate-determining step can be misleading. While the apparent activation energy is an enthalpy difference, simply examining the potential energy along the reaction pathway can be misleading in terms of both mechanistic insights and catalytic trends. It is somewhat more accurate to use the framework of the energetic span model,^73,74^ which suggests that the states with the lowest and highest free energy are kinetically relevant. Note that the overall reaction energy must be accounted for if the highest energy state comes before the lowest energy state. For heterogeneous catalysis, the energetic span model in its usual form can also be misleading in some cases as it usually assumes a linear mechanism. Thus, it is more accurate to use MKM to predict rates and analyze the mechanism using the degree of rate control or some other method.^75,76^

### 4.2 Approximations and Pitfalls That Require Additional Effort to Address

Some common approximations for catalytic systems can be feasibly relaxed with some additional computational effort by leveraging well-established methodologies. This will typically lead to improved accuracy and reliability in predicting catalytic behavior at the cost of reducing the number of materials that can be screened. Since it is rarely possible to relax all of these approximations simultaneously, it is important to consider which approximations are most important to address, and which are unlikely to significantly affect the accuracy.

Above, we discussed the use of simple stability tests to choose which surface configuration to study (Section 4.1); more accurate predictions of the surface structure under reaction conditions takes significantly more effort, although the surface structure can have large impacts on catalytic performance. Experimental studies highlight the significance of surface segregation and ensemble sizes in alloy surfaces. For example, surface segregation has been observed in oxygen reduction reaction conditions.^77^ Moreover, recent studies on dilute metal alloy nanomaterials^78^ demonstrate how gas pretreatment drives Pd segregation to the surface in dilute Pd-in-Au alloy catalysts of various concentrations. Specifically, the surface Pd content and its effect on ensemble size has a critical effect on hydrogen dissociation.^79^ Computational studies often simply assume an alloy structure, or perform a very simple test to predict the stable state. While these can be useful for fundamental understanding or for initial screening, and can bring some insight into what the likely state of the surface is under reaction conditions, caution should be exercised when applying the results to design for experimental testing. In some cases, a strong understanding of similar, existing systems can mitigate the risk.

Surface defects and steps can play a crucial role in catalytic processes, yet their effects are often not included in computational screening studies for alloys. The importance of steps and defects have been shown in specific systems; for example, studies of site-specific reactivity of atomic oxygen adsorbed at step defects on the Cu(110) surface during ammonia dehydrogenation reactions revealed higher reactivity at the top and bottom of {110} steps and the bottom of {001} steps, while minimal reactivity occurs at the top of {001} steps.^80^ Similarly, studies on the dry dehydrogenation of alcohols on Cu surfaces^81^ highlighted the catalytic potential of surface defects in facilitating reaction. While studying all surface facets and all possible defects is usually infeasible for design or screening, focused tests of how defects affect the performance of promising candidates can be useful, and it should be considered whether experimental trends will be dominated by terrace sites or defect sites.

When studying metal overlayers, it is common to model the overlayer as being in registry with the underlying crystal structure. However, unless the metals have very similar lattice constants, they are very likely to form a Moiré reconstruction, where the overlayer takes on a different unit cell than the underlying metal, resulting in periodic superstructures. These reconstructions can alter the surface properties and reactivity,^82^ analogous to effects seen for heterostructures of two-dimensional (2D) materials.^83^ The true reconstruction can be very complex and infeasible to model, but a smaller approximation is often achievable, and tools have been created to aid in this.^5^ However, for metal overlayers this framework will typically need to assume that the lattice constant of the bulk metal also applies to the overlayer. Overall, while a perfect model of the experimental overlayer system is not always achievable, using a more accurate model is likely to give more accurate results.

### 4.3 Approximations and Pitfalls That Are Very Challenging to Address

Some effects are quite difficult to capture with computational methods, and although they can generally be studied for individual systems it is often infeasible to include them in a design or screening study. Nevertheless, it is important to be aware of these approximations when designing for experiments.

Adsorbate–adsorbate interactions on surfaces can have a large effect on surface chemistry phenomena, as highlighted by recent studies. Adsorbate–adsorbate interactions on metal catalysts can be straightforwardly addressed for specific systems using existing methods, as long as there are not too many relevant intermediates to include, although there are additional complexities for oxide catalysts.^84,85^ For instance, a study of the water–gas shift reaction showed repulsive interactions between CO and other reaction intermediates.^86^ Similarly, computational simulations on Ru(0001) showed that adsorbate–adsorbate interactions influence NH_3_ decomposition, shifting the rate-determining step from N_2_ desorption to NH_2_* decomposition.^87^ Furthermore, the development of a machine learning-based hierarchical screening workflow has facilitated the estimation of surface adsorption structures for complex heterogeneous surface reactions.^88^

Accurate and efficient incorporation of coverage dependence into first-principles kinetic models is often quite challenging in a screening context,^89^ due to the huge number of different possible local environments for each intermediate and transition state. One approach is the utilization of a DFT-parametrized cluster expansion. This approach has proved useful in predicting rates and other kinetic parameters, with successful applications in catalytic NO oxidation on Pt(111) surfaces.^90^ Various strategies have been tested to see how adsorbate–adsorbate interactions affect CO adsorption and desorption.^91^ However, these types of approaches are often too intensive to use directly in screening or design. While it is feasible to include some adsorbate–adsorbate interactions in design studies by calculating or predicting the energetics in the presence of a coadsorbed species, a detailed account of the effect of adsorbate–adsorbate interactions across many surfaces is often impractical.

Incorporating nonadiabatic effects, particularly electronic excitation^92^ and associated electronic friction, is often very challenging as standard DFT codes cannot account for these effects. However, they have been shown to have impacts on surface chemistry. Examining the role of electronic excitations in N_2_ and H_2_ dissociation on Ru nanoparticles revealed the significant impact of electronic excitations on surface chemistry processes.^93^ Employing nonadiabatic dynamical calculations based on real-time, time-dependent DFT, it was observed that energy dissipation into electronic excitation surpasses thermal dissipation into phonons at short time scales. Excitation was found to elevate reaction barriers by ∼0.2 eV for N_2_, while excitations induced by one molecule can influence others. A separate study used molecular dynamics with electronic friction to study the dissociative adsorption and scattering of H_2_ on Ru(0001). The results indicate that while dissociative sticking probabilities are not heavily impacted by electronic friction under most conditions, electronic friction plays a crucial role in the inelastic scattering and energy distribution of reflected molecules.^94^ These findings suggest that metal nanoparticles and surfaces do not always remain in the ground state during reactions, potentially altering surface chemistry outcomes. These effects are often hard to capture, but can affect predicted quantities and could be particularly important when performing benchmark calculations.

**Figure 5:**
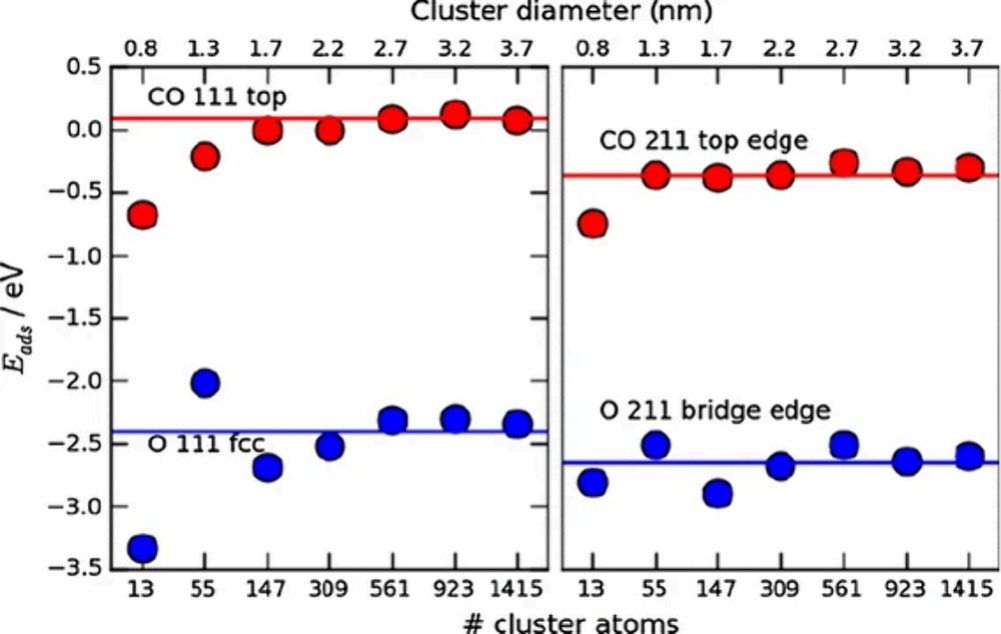
Adsorption energies of CO and O on the {111} facet and at the edge of fixed-geometry clusters as a function of cluster size. The horizontal lines denote the adsorption energies for {111} and {211} slab surface calculations. Adapted with permission from ref (95). Copyright 2011 Springer Nature.

Heterogeneous catalysts are often nanoparticles, but DFT studies usually model them as extended surfaces. For larger particles, this is likely a good approximation, especially if calculations for steps, defects, and various surface facets are combined to model the particle. However, nanoparticle structure and finite size effects could be important in some cases and are often difficult to model directly, particularly near the transition from molecular-like to bulk-like behavior. One study indicated that, for O and CO on gold nanoparticles, the adsorption energies became very similar to the bulk surfaces for clusters larger than approximately 560 atoms (2.7 nm; see Figure 5).^95^ Other work examining the finite-size effects of Pt clusters revealed that the surface catalytic properties converge to the single crystal limit for relatively small Pt clusters, with faster convergence than Au clusters.^96^ The difference was attributed to the presence of a partially filled d-shell in Pt. Similarly, research focusing on rutile titania nanoparticles has indicated that electronic finite size effects become negligible for nanoparticles larger than a certain threshold (>10 Å), except for the electronic gap and density of states which remain sensitive to size and shape.^97^ Another study indicated that finite-size effects are negligible for certain surface atoms in AuPd nanoalloys even for just 38-atom clusters.^98^ On another front, kinetic Monte Carlo simulations based on DFT shed light on selective acetylene hydrogenation over single-atom alloy nanoparticles. The study revealed a distinct reaction mechanism for Pd/Cu systems compared to extended surfaces, showing the possibility for nanoparticle morphology to enhance selectivity.^99^ Thus, the size at which clusters begin to behave similarly to bulk surfaces varies depending on the system.

For some systems, diffusion between different sites on surfaces or nanoparticles can affect reactivity. One computational study of CO oxidation over Pt nanoparticles revealed that different particle shapes lead to significantly different catalytic activities, which was attributed to kinetic interactions between sites.^100^ Additionally, for oxidation on Au and Au–Ag surfaces, it has been suggested that O_2_ dissociation may occur at bimetallic step sites, followed by diffusion of surface O and subsequent oxidation at other sites.^101^

Support effects can play a crucial role in modulating the catalytic activity and selectivity of supported metal catalysts; however, accurately capturing these effects is often difficult due to the complex nature of the interface and large system sizes. In DFT studies, metal–support interfaces are often modeled using quite simplified representations, with the metal represented as small particles/clusters or a narrow rod. For Au nanoparticles supported on TiO_2_, DFT calculations have indicated that cluster size significantly affects adsorption, with the {001} facet exhibiting stronger metal–support interaction. The presence of O-vacancy defects impacted adsorption but had minimal effect on H_2_ dissociation.^102^ Investigation into the catalytic performance of Ni nanoparticles supported on MgAlO_*x*_/ZrO_2_ hierarchical supports for the dry reforming of methane exhibited a particularly complex support that required layer matching and simplification of the Ni structure.^103^ Another study explored support effects in Pt-group metal catalysts supported on ceria and zirconia, focusing on NO reduction. Zirconia effectively lowered the transition state energy for NO dissociation compared to ceria, correlating with the observed NO reduction activity in experiment.^104^ A study into H_2_ oxidation kinetics over Au/TiO_2_ and Au/Al_2_O_3_ catalysts provided evidence for heterolytic H_2_ activation at the metal–support interface.^105^ Overall, these studies show the multifaceted nature of support effects in catalysis. Capturing these effects computationally typically requires significant simplification of the system, and is still usually too computationally intensive for effective screening.

Accurate estimation of entropy to calculate free energies is another important issue in understanding surface chemistry and catalysis, as shown in recent studies. While the harmonic approximation is straightforward to apply and is often reasonably effective, several studies have indicated it is often not quantitatively accurate. For instance, investigation into the decomposition of CHO on Rh(111) proposed a machine-learning approach to efficiently estimate free energy barriers in catalytic reactions.^106^ One investigation compared standard analytical models used to estimate adsorption free energies with numerical evaluations derived from translational Schrödinger equations, showing significant variations across methods.^107^ Assessing rate constant parameters for formate dehydrogenation on Au(110) and Cu(110) surfaces revealed higher pre-exponential factors for CO_2_ formation due to increased entropy in monodentate transition states versus bidentate configurations.^108^ In another study, various entropy approximations were compared for CO oxidation over Pt(111) surfaces. The complete potential energy sampling method emerged as the preferable choice, showing the best agreement with experimental data.^109^

Accurately accounting for solvent effects in aqueous-phase reactions can also be very challenging. Solvents can influence reaction rates and product selectivity in a number of ways, including by directly participating in the reaction steps, (de)stabilizing intermediates or transition states, competing for active sites, etc. They can also exhibit less direct influences, such as modifying active sites or altering the structure or stability of catalysts.^110^ While advances in computational chemistry have led to the development of multiple techniques for including solvent effects, a generally applicably strategy has yet to emerge.^110^ In some cases, a few water molecules (sometimes called microsolvation) or continuum solvation^111,112^ approaches can be effective, but these are not accurate in all situations. MD approaches are more accurate in principle, but show a strong dependence on the starting geometry for the time scales that are generally accessible with DFT, indicating it is challenging to reach equilibrium.^111^ Thus, while solvation effects should be included as accurately as is feasible for a given study, a completely general, accurate, and computationally tractable method has not yet emerged, and solvation methods remain an area of active research.

Finally, for electrochemical reactions, the effect of the applied electric potential can be difficult to accurately capture. Applying a uniform field can bring some insight, but may not accurately represent the surface environment, and it can be difficult to associate a field with a specific macroscopic potential. Various methods for including the potential by charging the surface have been developed,^113−115^ which can improve the realism of the model, but a completely realistic model of the electrochemical interface is very challenging, partly due to the interaction of the electric potential and the solvent. The electric double-layer is very complex, with interactions between solvent, electric field, catalyst, adsorbed species, and ions, and no single approach can effectively solve this complex problem. Recent reviews have given much more comprehensive discussions of solvent and electrochemical effects.^116−118^

The importance of all of these approximations depends strongly on the system under study. For example, particle size effects can be important for very small particles but are usually relatively unimportant for the relatively large particles used in many catalytic systems. In general, making some attempt to estimate stability under reaction conditions is crucial for developing a correspondence between computation and experiment, and some consideration of adsorbate–adsorbate interactions should be undertaken where possible as they are very common and have the potential to significantly impact reaction energetics.

## 5 Conclusions

The studies discussed here showing successful computational design with experimental validation show that computation can be a powerful tool in developing new catalysts. As compared to traditional approaches in catalyst design, computation can point to materials that may not be intuitively obvious, can more efficiently narrow down the design space, and can lead to greater understanding of the function of the designed catalyst.

It is crucial to understand the approximations for a given computational design workflow and judiciously choose the workflow that is most suited for a given design task. All computational studies require some level of approximation in order to be feasible; thus, the estimated accuracy, computational requirements, and difficulty of experimental testing each material must all be weighed against each other. Activity, selectivity, stability, synthesizability, and cost may all be factors to consider in design, and each will have its own level of approximation. Furthermore, different computational strategies are often required when attempting to design for experimental validation as compared to understanding an existing catalytic system or developing design methodologies.

In general, more sophisticated screening brings a higher computational cost but often leads to a higher likelihood of accurately predicting high-performing catalysts. In the end, because of the approximations that are still necessary, computation is often a guide toward regions of the design space that are likely to contain promising materials, rather than an exact recipe for a final catalyst. Additionally, it is nearly always more effective to combine computation with researcher judgment based on a strong understanding of catalysis science. This understanding can be used to choose the proper design space to screen and the proper design methodology and approximations for a given system, and to prioritize the promising candidates identified by computational screening.
